# Space-by-time decomposition for single-trial decoding of M/EEG activity

**DOI:** 10.1016/j.neuroimage.2016.03.043

**Published:** 2016-06

**Authors:** Ioannis Delis, Arno Onken, Philippe G. Schyns, Stefano Panzeri, Marios G. Philiastides

**Affiliations:** a Institute of Neuroscience and Psychology, University of Glasgow, Glasgow, G12 8QB, United Kingdom; b Department of Biomedical Engineering, Columbia University, New York, NY 10027, USA; c Neural Computation Laboratory, Center for Neuroscience and Cognitive Systems@UniTn, Istituto Italiano di Tecnologia, Via Bettini 31, 38068, Rovereto (TN), Italy

**Keywords:** M/EEG, NMF, Single-trial analysis, Neural representation, Dimensionality reduction, Decoding

## Abstract

We develop a novel methodology for the single-trial analysis of multichannel time-varying neuroimaging signals. We introduce the space-by-time M/EEG decomposition, based on Non-negative Matrix Factorization (NMF), which describes single-trial M/EEG signals using a set of non-negative spatial and temporal components that are linearly combined with signed scalar activation coefficients. We illustrate the effectiveness of the proposed approach on an EEG dataset recorded during the performance of a visual categorization task. Our method extracts three temporal and two spatial functional components achieving a compact yet full representation of the underlying structure, which validates and summarizes succinctly results from previous studies. Furthermore, we introduce a decoding analysis that allows determining the distinct functional role of each component and relating them to experimental conditions and task parameters. In particular, we demonstrate that the presented stimulus and the task difficulty of each trial can be reliably decoded using specific combinations of components from the identified space-by-time representation. When comparing with a sliding-window linear discriminant algorithm, we show that our approach yields more robust decoding performance across participants. Overall, our findings suggest that the proposed space-by-time decomposition is a meaningful low-dimensional representation that carries the relevant information of single-trial M/EEG signals.

## Highlights

• Space-by-time decomposition for multichannel time-varying signal analysis.
• Extraction of spatial and temporal components of single-trial M/EEG activity.
• Full and succinct characterization of EEG data during a visual categorization task.
• Single-trial decoding based on task-relevant features.
• Robust and consistent decoding results across participants.

## Introduction

Non-invasive electrophysiological neuroimaging techniques, such as magneto/electroencephalography (M/EEG), record brain activity across time (with high temporal resolution) at multiple locations (with good spatial specificity), providing signals that can potentially elucidate the spatial and temporal dynamics of neural information processing. In an experiment, M/EEG activity is typically recorded over multiple repetitions (or trials) that may differ in the type of stimulus presented, in the task the subject is asked to perform or the subject's response, or in other parameters collectively defining the experimental conditions. Hence, in order to analyse M/EEG recordings, the data can be represented as three-dimensional arrays indexed by space (sensors), time and trials (including all experimental conditions).

To describe such data, different methods have been proposed in the literature. The simplest approach is to average the recordings across trials at any location to compute Evoked Response Potentials (ERPs). However, the ERPs disregard the single-trial information that the brain processes to make decisions and produce behaviour (Pernet et al., 2011, Quian Quiroga and Panzeri, 2009, Sajda et al., 2009). More recently, a variety of multivariate analysis techniques aiming to identify patterns of neural activity from single-trial M/EEG data have been developed (Cichy et al., 2014, Panzeri et al., 2015, Parra et al., 2008). In this regard, a popular approach builds upon the application of dimensionality reduction methods such as principal component analysis (PCA) (Friston et al., 1996) or independent component analysis (ICA) (Makeig et al., 1996). These techniques rely on assumptions such as orthogonality or independence of the extracted components that, however, may not be satisfied by the generators of neural activity. Importantly, such methods are usually applied after forming a single dimension, thereby decomposing only the spatial (or temporal) dimension of the data and missing out on decomposing the other (see, e.g., (Delorme and Makeig, 2004)). Another alternative is the use of decoding algorithms, such as linear discriminant analysis (LDA) (Parra et al., 2005), logistic regression (LR) (Gherman and Philiastides, 2015, Parra et al., 2002) and support vector machines (SVM) (Cichy et al., 2014). These methods typically decompose data in space at each time point or in short pre-defined temporal windows, thereby ignoring the full temporal profile of the data.

In this study, we develop a methodology for the decomposition of three-dimensional M/EEG signals into a series of distinct ‘components’ along each of the dimensions. We call the novel method we derive here space-by-time M/EEG decomposition. The space-by-time decomposition provides a compact representation of single trial M/EEG data describing where (spatial components), when (temporal components) and how much M/EEG signals are activated in each trial (scalar coefficients representing the relative contribution of each combination of spatial and temporal components). The key point of the proposed decomposition resides in the possibility to combine any of the temporal modules with any of the spatial ones, which leads to a low-dimensional, though flexible and functional representation of M/EEG activity patterns.

The space-by-time M/EEG decomposition is based on the use of non-negative matrix factorization (NMF) (Lee and Seung, 1999), a dimensionality reduction algorithm that imposes non-negativity constraints to the extracted components leading to a sparse representation of the data. This property of NMF and the lack of any other assumptions make it a natural method for identifying low-dimensional parts-based decompositions (Lee and Seung, 1999). NMF decompositions require non-negative input data, thus they cannot be used in their original form to properly describe M/EEG data that are characterized by sign variations over time reflecting important changes in neural activity (Ng et al., 2013, Panzeri et al., 2015, Whittingstall and Logothetis, 2009). Here we overcome this difficulty by using cluster-NMF (a variant of standard NMF that extends its application to data with negative entries (Ding et al., 2010)). Also, taking advantage of recent progress in analysis of muscle activation signals (Delis et al., 2014, Delis et al., 2015), we introduce a single-trial NMF decomposition that factors out simultaneously and separately the spatial and temporal dimension of single-trial M/EEG activity. Hence, we decompose M/EEG data into non-negative spatial and temporal components and use coefficients of arbitrary sign to combine them in single trials.

To illustrate the usefulness of the resulting space-by-time decomposition, we apply it to EEG activity recorded during a simple visual categorization task. As this EEG dataset has been studied thoroughly before (Lou et al., 2014, Philiastides et al., 2006, Philiastides and Sajda, 2006a, Philiastides and Sajda, 2006b, Ratcliff et al., 2009), the relevant EEG signatures are known and thus, it can be used as benchmark for testing and validating the proposed methodology. In the following, we first introduce the space-by-time decomposition and develop an algorithm based on NMF (Lee and Seung, 1999) that implements it. Then, we test our methodology on the benchmark dataset and compare its outcome with results obtained in previous studies using a different decoding approach. We finally discuss the main properties of the proposed methodology and its applicability to M/EEG data analysis.

## Materials and methods

### Outline of the proposed method

In this study, we propose a methodological framework for the single-trial analysis of M/EEG signals. Our approach is particularly useful for studies that aim to extract information from M/EEG data and in turn relate this information to condition differences. The method proceeds in two steps. Firstly, we use a dimensionality reduction algorithm to identify a small number of components of M/EEG activity and their projections to single-trial M/EEG signals, which we refer to as component activations. Secondly, we perform a decoding analysis to relate the extracted component activations with differences between experimental conditions. We detail these steps in the following sections.

### Space-by-time M/EEG decomposition

We introduce a method, termed space-by-time M/EEG decomposition, that decomposes single-trial M/EEG activity into a small number of spatial and temporal components (Delis et al., 2014). In order to approximate the M/EEG signals recorded from *S* sensors over *T* time frames, the space-by-time decomposition identifies distinct spatial and temporal components and combines them in single trials using scalar coefficients. Formally, the M/EEG activity **M**_*n*_ with dimensions (*T* × *S*) recorded during one trial *n* is factorized as follows:

(1) $$M_{n}≃W_{\mathrm{tem}}H_{n}W_{\mathrm{spa}},\forall n\in \left\{1,...,N\right\}$$

where **W**_*tem*_ is a (*T* × *P*) matrix whose columns are the temporal components, **W**_*spa*_ is a (*L* × *S*) matrix whose rows are the spatial components and **H**_*n*_ is a (*P* × *L*) matrix containing the coefficients that combine each one of the *P* temporal components with each one of the *L* spatial ones. The number of the temporal and spatial components (*P*, *L* respectively) are free parameters of the analyses. Here we chose them from the data using a decoding approach (see section Selecting the number of dimensions below). The product *PL* is the dimension of the linear space on which each single-trial activity **M**_*n*_ is represented and the dimensionality reduction is effective if *PL* ≪ *TS*.

The tri-factorization in Eq. (1) can be rewritten as the following double summation:

(2) $$m_{n}\left(t\right)≃\sum_{i=1}^{P}\sum_{j=1}^{L}w_{\mathrm{tem}}^{i}\left(t\right)h_{n}^{i,j}w_{\mathrm{spa}}^{j}$$

where temporal and spatial components are now given in vector forms as **w**_*tem*_^*i*^, **w**_*spa*_^*j*^ respectively and *h*_*n*_^*i* , *j*^ is a scalar activation coefficient combining components *i* and *j* in trial *n*.

### Component extraction algorithm

To implement the above decomposition, we develop a component extraction algorithm based on Non-negative Matrix Factorization (NMF) (Lee and Seung, 1999). NMF is a dimensionality reduction method that aims to learn part-based representations of the input data by imposing non-negativity constraints on the extracted components and their activations.

In our formulation, another constraint (except for non-negativity) is that the matrices **W**_*tem*_, **W**_*spa*_ representing the temporal and spatial components respectively must be trial-independent, i.e. invariant across trials and conditions. The objective of the algorithm is to find **W**_*tem*_, **W**_*spa*_ together with the set of matrices **H** = (**H**_*n*_)_*n* ∈ {1, ... , *N*}_ such that they minimize the total reconstruction error $E^{2}=\sum_{n}E_{n}^{2}$, where *E*_*n*_^2^ is defined as the squared Frobenius norm of the single-trial approximation error:

(3) $$E_{n}^{2}=||M_{n}-W_{\mathrm{tem}}H_{n}W_{\mathrm{spa}}||{}^{2}=\sum_{i,j}{\left(M_{n}^{i,j}-{\left(W_{\mathrm{tem}}H_{n}W_{\mathrm{spa}}\right)}^{i,j}\right)}^{2}.$$

For non-negative data matrices **M**_*n*_, this decomposition is also known as non-negative Tucker-2. Optimal algorithms implementing this decomposition have been derived before (Kim and Choi, 2007, Morup et al., 2007, Phan and Cichocki, 2010). In the Appendix A, we detail one of these non-negative decomposition algorithms termed sample-based non-negative matrix tri-factorization (sNM3F) (Delis et al., 2014).

Here, however, as we are dealing with datasets that also contain negative entries we need to introduce a variant of the original sNM3F factorization to deal with this issue. We do so by combining sNM3F with cluster-NMF, a variant of standard NMF that extends its application to data with negative entries (Ding et al., 2010).

In particular, cluster-NMF extracts non-negative components **W** that approximate a signed data matrix **M** as follows:

(4) $$M≃MW^{T}W$$

The above approximation becomes equality when matrix **W** is orthogonal. This orthogonality objective makes **W** sparse and enhances its clustering ability.

To minimize the squared Frobenius norm of the approximation error *E*^2^ = ||** M** − **MW**^*T*^**W** ||^2^, the algorithm iteratively estimates **W** using the multiplicative update rule:

(5) $$W^{i,j}\leftarrow W^{i,j}\sqrt{\frac{{\left[{\left(M^{T}M\right)}^{+}W^{T}\right]}^{i,j}+{\left[W^{T}W{\left(M^{T}M\right)}^{-}W^{T}\right]}^{i,j}}{{\left[{\left(M^{T}M\right)}^{-}W^{T}\right]}^{i,j}+{\left[W^{T}W{\left(M^{T}M\right)}^{+}W^{T}\right]}^{i,j}}}$$

where we separate the positive and negative parts of a matrix **A** as **A**_*i* , *j*_^+^ = (|** A**_*i* , *j*_ | + **A**_*i* , *j*_)/2 and **A**_*i* , *j*_^−^ = (|** A**_*i* , *j*_ | − **A**_*i* , *j*_)/2.

Hence, here we exploit the ability of cluster-NMF to handle signed data and adapt it to the tri-factorization introduced by the space-by-time decomposition. In particular, we keep the temporal **W**_*tem*_ and spatial components **W**_*spa*_ non-negative so that they represent clusters in time and space respectively, whereas we allow the activation coefficients **H**_*n*_ to take negative values. Hence, in our formulation, the single-trial information in the signed EEG data is captured by the signed single-trial coefficients that combine the components.

Importantly, cluster-NMF has all the advantages of NMF, i.e. yielding low-dimensional parts-based representations of the data, and also identifies components that are naturally sparse and correspond to distinct data clusters, which makes them easily interpretable. Thus, the new algorithm we developed here (termed scNM3F, i.e. sample-based cluster non-negative matrix tri-factorization) performs a concurrent estimation of distinct non-negative spatial and temporal components (like sNM3F) and also inherits the properties of cluster-NMF, i.e. applicability to signed data and also sparsity and clustering.

Based on the update rules of sNM3F and cluster-NMF, we derived iterative update rules for scNM3F. We apply cluster-NMF to iteratively update **W**_*tem*_ and **W**_*spa*_ using the following rules:

(6) $$W_{\mathrm{tem}}^{i,j}\leftarrow W_{\mathrm{tem}}^{i,j}\sqrt{\frac{{\left[{\left(M_{\mathrm{tem}}M_{\mathrm{tem}}^{T}\right)}^{+}W_{\mathrm{tem}}\right]}^{i,j}+{\left[W_{\mathrm{tem}}W_{\mathrm{tem}}^{T}{\left(M_{\mathrm{tem}}M_{\mathrm{tem}}^{T}\right)}^{-}W_{\mathrm{tem}}\right]}^{i,j}}{{\left[{\left(M_{\mathrm{tem}}M_{\mathrm{tem}}^{T}\right)}^{-}W_{\mathrm{tem}}\right]}^{i,j}+{\left[W_{\mathrm{tem}}W_{\mathrm{tem}}^{T}{\left(M_{\mathrm{tem}}M_{\mathrm{tem}}^{T}\right)}^{+}W_{\mathrm{tem}}\right]}^{i,j}}}$$

(7) $$W_{\mathrm{spa}}^{i,j}\leftarrow W_{\mathrm{spa}}^{i,j}\sqrt{\frac{{\left[{\left(M_{\mathrm{spa}}^{T}M_{\mathrm{spa}}\right)}^{+}W_{\mathrm{spa}}^{T}\right]}^{i,j}+{\left[W_{\mathrm{spa}}^{T}W_{\mathrm{spa}}{\left(M_{\mathrm{spa}}^{T}M_{\mathrm{spa}}\right)}^{-}W_{\mathrm{spa}}^{T}\right]}^{i,j}}{{\left[{\left(M_{\mathrm{spa}}^{T}M_{\mathrm{spa}}\right)}^{-}W_{\mathrm{spa}}^{T}\right]}^{i,j}+{\left[W_{\mathrm{spa}}^{T}W_{\mathrm{spa}}{\left(M_{\mathrm{spa}}^{T}M_{\mathrm{spa}}\right)}^{+}W_{\mathrm{spa}}^{T}\right]}^{i,j}}}$$

which minimize the respective approximation errors:

(8) $$E_{\mathrm{tem}}^{2}=||M_{\mathrm{tem}}-W_{\mathrm{tem}}W_{\mathrm{tem}}^{T}M_{\mathrm{tem}}||{}^{2}$$

(9) $$E_{\mathrm{spa}}^{2}=||M_{\mathrm{spa}}-M_{\mathrm{spa}}W_{\mathrm{spa}}^{T}W_{\mathrm{spa}}||{}^{2}$$

**M**_*tem*_ and **M**_*spa*_ are reshaped versions of the input matrix **M** with dimensions (*T* × *SN*) and (*TN* × *S*) respectively. Use of multiple objective functions has been proposed before in the context of non-negative Tucker decompositions (Cichocki et al., 2009). Importantly, these rules update the spatial and temporal components using both the positive and the negative entries of the input data matrix **M**. This stands in contrast to most algorithms that extract non-negative components using a half-wave rectification of the input matrix, which ignores the negative entries (Phan and Cichocki, 2008, Phan and Cichocki, 2011). In Supplementary Material, we present an illustrative example of this difference on simulated data (Supp. Figs. 4-5).

**H**_*n*_ is iteratively updated for all *n* ∈ {1, ... , *N*} to minimize the single-trial approximation error (Eq. (3)) as follows:

(10) $$H_{n}\leftarrow W_{\mathrm{tem}}^{-1}M_{n}W_{\mathrm{spa}}^{-1}$$

where **W**_*tem*_^− 1^, **W**_*spa*_^− 1^ represent pseudoinverses of the two matrices. Replacing pseudoinverses with transposes in Eq. (6) gave highly similar results, which indicates that the matrices **W**_*tem*_, **W**_*spa*_ become nearly orthogonal (as is the objective of cluster-NMF, see Appendix A) from the first iterations in the update procedure. This observation supports the robust convergence of the algorithm.

Ultimately, the scNM3F algorithm takes the following form:

1) Initialize **W**_*tem*_(*T* × *P*), **H**(*P* × *LN*), and **W**_*spa*_(*L* × *S*) with random entries
2) Given **H** and the data matrix **M**(*T* × *S* × *N*),
  a. Reshape **M** → **M**_*spa*_(*TN* × *S*)
  b. Update **W**_*spa*_ using Eq. (5).
  c. Reshape **M** → **M**_*tem*_(*T* × *SN*).
  d. Update **W**_*tem*_ using Eq. (4).
3) Given **W**_*tem*_ and **W**_*spa*_:
  a. For all *n* ∈ {1, ... , *N*}, update **H**_*n*_ using Eq. (6).
4) If decrease in approximation error $\sum_{n=1}^{N}{\left\|M_{n}-W_{\mathrm{tem}}H_{n}W_{\mathrm{spa}}\right\|}^{2}$ is below a given tolerance, stop. Otherwise, go to step 2.

An open-source Matlab software implementation of scNM3F is made available online at https://sites.google.com/site/ioannisdeliswebpage/software/scNM3F.zip.

Although convergence of this algorithm cannot be proved formally because it uses more than one objective function, when we applied it to the EEG data, it always showed good convergence. The single-trial approximation error decreased at each iteration until reaching a plateau, when the algorithm stopped. Importantly, as we demonstrate in the Results section, the output of the algorithm comprised meaningful EEG components with distinct functional roles that carried information about differences in experimental conditions.

### Component clustering

To compare components of the same type (spatial or temporal) extracted from different subjects, we grouped them using an agglomerative hierarchical cluster analysis (Hastie et al., 2009). In the following, we will present the procedure in detail for spatial components, but the same procedure was followed also for clustering the temporal components. We first assessed whether the spatial components we extracted from different subjects contained similar sensor activations. To do this, we considered spatial components as *S*-dimensional vectors and used as measure of similarity the correlation coefficient. We computed correlation coefficients (*r*_*i* , *j*_) between all pairs of components (*i*,*j*) across all pairs of subjects. Then, the clustering algorithm linked all components based on their similarity and created a hierarchical cluster tree (Matlab function “linkage” with the “average” distance method, i.e., using as distance between two clusters the average distance between all pairs of objects across the two clusters). We partitioned the tree with the minimum number of clusters for which there was no more than one component from the same subject in each cluster. In this way, we grouped together components that had the highest similarity (correlation coefficient) and ensured that each cluster did not contain more than one component from each subject. This procedure yielded three clusters of temporal components and two clusters of spatial components. For illustration, we represented each cluster by the average across all cluster members (see Fig. 2 for the averages of the spatial and temporal clusters).

### Decoding analyses

The single-trial coefficients **H**_*n*_ of the space-by-time decomposition encode the level of activation of the components in individual trials. Specifically, the coefficient *h*_*n*_^*k* , *m*^ represents the relative amplitude of temporal component *k* in the electrodes defined by spatial component *m* on trial *n.* Hence, if a particular temporal/spatial component exhibits different activation strengths depending on the experimental condition, these differences will be reflected on the values of the coefficients **H**_*n*_. Thus, these coefficients can be used as the single-trial parameters that relate each component to the stimulus presented or the task condition imposed on each trial. Hence, to test if the obtained space-by-time decompositions allow discrimination between experimental conditions, we employed a single-trial decoding analysis that used the coefficients **H**_*n*_ as decoding parameters. In particular, we used linear discriminant analysis (LDA) in conjunction with a leave-one-out cross-validation and quantified decoding accuracy as the area under the ROC curve (*A_z_*). This analysis allowed us to tease apart the contribution of each temporal/spatial component to stimulus/condition discrimination and determine the components or combinations of components that carry most information about stimulus/condition differences.

### Decoding performance significance test

To assess the significance of decoding performance, we employed a permutation test where we randomly shuffled the trial labels 500 times and computed discrimination performance. This permutation test ensured that the association between the stimuli and neural representations was abolished, while the distributions of single-trial coefficients were unaffected. We concluded that decoding performance was statistically significant if the obtained values were outside the 95% confidence intervals of these permutation distributions (Philiastides and Sajda, 2006a, Philiastides and Sajda, 2006b).

### Selecting the number of dimensions

In the literature, there are several approaches for determining the number of components in low-dimensional decompositions, such as DIFFIT and convex-hull (Ceulemans and Kiers, 2006, Cichocki et al., 2009, Timmerman and Kiers, 2000). In this study, our focus was on extracting components that relate to condition differences, i.e. components that decode different experimental parameters. For this reason, we used the decoding approach we described above for selecting the number of components of the space-by-time decompositions. This decoding criterion has been shown to reliably determine components that capture the condition-discriminating information and exclude condition-irrelevant variability (Delis et al., 2013a, Delis et al., 2013b). More precisely, we gradually increased the numbers of spatial and temporal components and computed face versus car discrimination performance of the resulting decompositions. We stopped when adding a (spatial or temporal) component did not give any significant gain (*p* > 0.05) in decoding (see Supp. Figure 1 for an illustration of this selection criterion on the data of one subject) (Delis et al., 2013b). Significance was computed using a permutation test similar to the one described above. In this case, the coefficients corresponding to the added component were randomly permuted while the distributions of all other coefficients were unaffected.

### Testing the method on EEG recordings

To test the effectiveness of the proposed method on real data, we applied it to the EEG recordings obtained during the performance of a simple visual categorization task. We chose this experimental dataset because the task is well established and has been used extensively in our lab using a more conventional sliding-window LDA approach (i.e. spatial decomposition over multiple time windows) (Philiastides et al., 2006, Philiastides and Sajda, 2006a, Philiastides and Sajda, 2006b, Philiastides and Sajda, 2007, Ratcliff et al., 2009). As such, the relevant neural signatures have been investigated intensively with more traditional methods and will serve as useful benchmark for testing the new method.

### Subjects

Ten subjects with normal or corrected to normal vision and no history of neurological problems provided informed consent and participated in the study. The experiment was conducted in accordance with the guidelines and approval of the Columbia University Institutional Review Board.

### Stimuli

12 face grayscale images (face database; Max Planck Institute for Biological Cybernetics, Tuebingen, Germany) and 12 car grayscale images (image size, 512 × 512 pixels; 8 bits/pixel) were used as visual stimuli for the experiment. All images were equated for spatial frequency, luminance, and contrast and had identical magnitude spectra (average magnitude spectrum of all images in the database). We used the weighted mean phase technique to manipulate phase spectra in order to generate a set of images characterized by their percentage of phase coherence. Each image was processed to have six different phase coherence values (20, 25, 30, 35, 40, and 45%).

### Behavioural paradigm

**Fig. 1:**
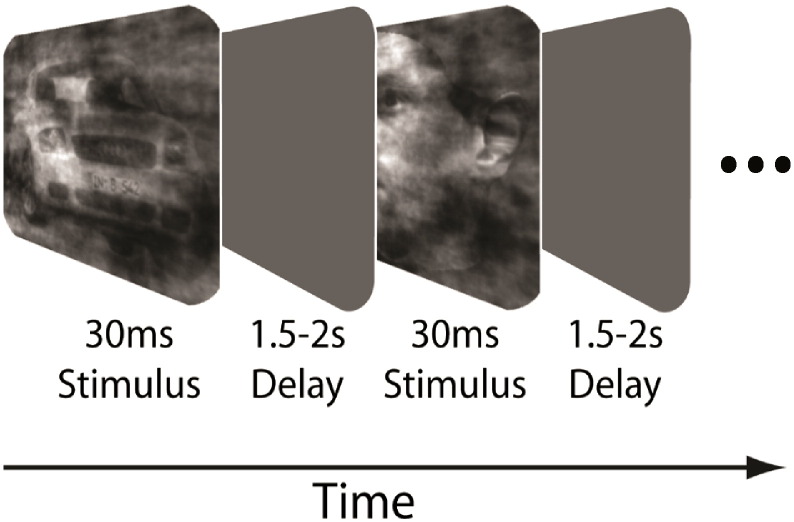
Schematic representation of the behavioural paradigm. Within a block of trials subjects were instructed to fixate on the centre of the screen and were subsequently presented, in random order, with a series of different face and car images at one of six phase coherence levels. Each image was presented for 30 ms, followed by an inter-stimulus interval lasting between 1500 and 2000 ms, during which subjects were required to discriminate among the two types of images and respond by pressing a button. A block of trials was completed once all face and car images at all six phase coherence levels have been presented.

Subjects performed a simple face-vs-car categorization task. A schematic representation of the behavioural paradigm is given in Fig. 1. Within a block of trials, face and car images over a range of phase coherences were presented in random order. We chose the range of phase coherence levels to obtain a full psychometric curve for each participant. At the beginning of a block of trials subjects fixated at the centre of the screen. Images were presented for 30 ms followed by an inter-stimulus-interval (ISI) which was randomized in the range of 1500–2000 ms. Subjects were instructed to respond as soon as they identified the type of image and before the next image was presented. Subjects reported their decision regarding the type of image by pressing one of two mouse buttons — left for faces and right for cars — using their right index and middle fingers respectively. A block of trials consisted of 24 trials of both face and car images at each of six different phase coherence levels, a total of 144 trials. There were a total of four blocks in each experiment. Trials where subjects failed to respond within the ISI were discarded from further analysis. More details about the experimental protocol can be found in (Philiastides et al., 2006, Philiastides and Sajda, 2006a, Philiastides and Sajda, 2006b).

### EEG data acquisition and preprocessing

We recorded EEG data simultaneously from 60 Ag/AgCl scalp electrodes and from three periocular electrodes placed below the left eye and at the left and right outer canthi. All channels were referenced to the left mastoid with input impedance < 15kOhm and chin ground. Data were sampled at 1000 Hz with an analogue pass band of 0.01–300 Hz using 12 dB/octave high pass and eighth-order Elliptic low pass filters. Subsequently, DC drifts were removed using a software based 0.5 Hz high pass filter and 60 and 120 Hz (harmonic) notch filters were applied to minimize line noise artefacts. Subjects also performed an eye muscle calibration experiment in order to determine linear components associated with eye blinks and saccades (using principal component analysis). These components were subsequently projected out of the EEG recorded during the main experiment. Trials with strong eye movement or other movement artefacts were manually removed by inspection. Ultimately, there were at least 40 artefact-free trials for any given condition (i.e. at least 80 trials for both sets of face and car trials at each phase coherence level). For all further analyses, we used the resulting EEG signals from all (*S* = 60) electrodes in the time period starting 100 ms before the onset of stimulus presentation and ending 600 ms after it (*T* = 700 timeframes for each of the *N* trials). Thus, we built a (*T* × *S* × *N*) three-dimensional matrix **M** from the EEG data of each subject and used it as input to the space-by-time decomposition.

### Performance comparison with standard decoding approaches

To compare our approach with more conventional sliding-window decoding schemes used in previous studies (Philiastides et al., 2006, Philiastides and Sajda, 2006a, Philiastides and Sajda, 2006b), we also performed a sliding-window LDA analysis on the original EEG signals (without any dimensionality reduction). We used a Fisher discriminant to estimate an *N*-dimensional spatial weighting vector for discriminating between two conditions over specific temporal windows. To allow direct comparisons with the space-by-time representations, we centred the temporal windows at the peaks of the temporal components identified by scNM3F and set their duration to 60 ms. Decoding performance was obtained in an identical manner to the one described above (i.e. area under an ROC curve, using leave-one-out cross validation).

## Results

### Behavioural performance

All subjects performed the face versus car categorization task nearly perfectly at the highest phase coherence (96.8 ± 1.6%; average ±* SEM* across subjects). Their performance dropped with difficulty and was near chance at the lowest coherence (58 ± 2.8% on average). Reaction times were shorter at the highest coherence (570 ± 51 *ms* on average) and longer at the lowest (676 ± 72 *ms* on average). Overall phase coherence level was positively correlated with categorization accuracy (*p* = 0.0003 , *t*(*df* = 60) = 11.30), and negatively correlated with reaction time (*p* = 0.0004 , *t*(60) = − 10.58), indicating that the phase coherence level of the stimulus had a strong effect on subjects' perceptual choices.

### Space-by-time decomposition

**Fig. 2:**
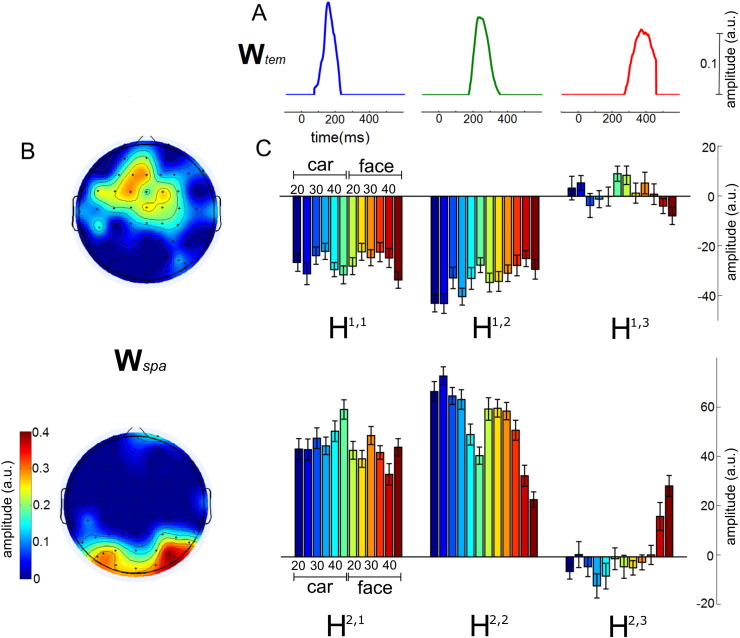
Space-by-time decomposition of EEG data of an example subject. A) The three temporal components with consecutive peaks found by the scNM3F algorithm to describe the EEG data. B) The two spatial components: one comprises mainly occipital electrodes and the other mainly centro-frontal electrodes. C) The 6 (2 × 3) coefficients combining the three temporal with the two spatial components trial-averaged for each stimulus (face or car) and at each phase coherence level (20, 25, 30, 35, 40, and 45%).

To gain insights about the space-by-time decomposition, we first illustrate its output for a representative subject. When applied to the single-trial EEG data of this subject, the space-by-time decomposition identified three temporal components **W**_*tem*_ with successive single peaks (Fig. 2A) and two spatial components **W**_*spa*_ activating different sensors (centro-frontal and occipitoparietal electrodes respectively) (Fig. 2B). To approximate the original EEG signals in single trials, the algorithm identifies 2 × 3 = 6 single-trial coefficients **H**_*n*_ that combine the three temporal with the two spatial components. Fig. 2C shows these combination coefficients trial-averaged for each stimulus and phase coherence level, in order to illustrate their modulations by both trial type (face or car) and phase coherence level. In particular, the combination of the first temporal component with the second (occipitoparietal) spatial one (H^2 , 1^) appeared to discriminate faces from cars for the high phase coherence level trials (bottom left in Fig. 2C). Activations of the second temporal component depended instead on the phase coherence levels. In particular, higher phase coherence level trials had lower negative activations of the second temporal component in the centro-frontal areas of the first spatial component (H^1,2^, top middle in Fig. 2C) and lower positive activations in the occipitoparietal areas of the second spatial component (H^2,2^, bottom middle in Fig. 2C). Similar to the first temporal component, the third also served to discriminate faces from cars in both the centro-frontal and the occipital components. In particular, in high coherence phase trials, the third temporal component was activated negatively in the centro-frontal areas (H^1,3^, top right in Fig. 2C) and positively in the occipital areas (H^2,3^, bottom right in Fig. 2C).

### Spatial and temporal neural components

**Fig. 3:**
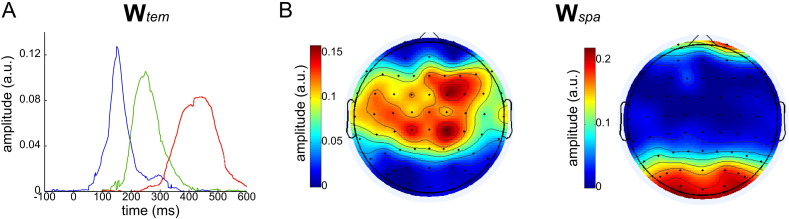
Temporal and spatial components clustered and averaged across all ten subjects. A) The three temporal components have consecutive single peaks at 150 ms, 250 ms and 450 ms respectively. B) The two spatial components illustrated as scalp maps of electrode activation levels. The first spatial component comprises mainly activations of centro-parietal, central and centro-frontal electrodes. The second spatial component comprises high activations of occipital electrodes and lower activations of parieto-occipital and frontopolar electrodes.

To test the robustness of the above findings, we applied the method to EEG data recorded from all ten participants of the experiment to uncover the underlying temporal and spatial structure in all recorded datasets. We found three temporal components in four of the subjects and two temporal components in the remaining six subjects. We clustered the temporal components across subjects using agglomerative hierarchical clustering (see Materials and Methods for details) and found three clusters that include components with different timings. The temporal components averaged across subjects for each cluster are shown in Fig. 3A. The first temporal component (blue curve) was present in 5 subjects, the second temporal component (green curve) was found in 9 subjects and the third temporal component (red curve) was present in all 10 subjects. On average, the first temporal component peaked at about 150 ms and was followed by a second one with a peak about 100 ms later and a third one whose onset was at about 300 ms and had a flat peak at about 450 ms. Regarding the spatial structure, all ten datasets returned two spatial components that were clustered using agglomerative hierarchical clustering (see Materials and Methods for details) and averaged across subjects as shown in Figs. 3B-C. The first spatial component had centroparietal, central and centrofrontal activations (Fig. 3B) and the second one had high occipitoparietal and frontopolar activations (Fig. 3C). These component topographies are consistent with the ones found in our example subject. Overall, both in space and time, the components determined by our algorithm are in perfect agreement with previous studies on the same dataset that reported such spatial and temporal structure (Philiastides et al., 2006, Philiastides and Sajda, 2006a, Philiastides and Sajda, 2006b). We note here that all spatial and temporal components are depicted with positive values, however their actual sign and strength on individual trials is determined by the (mixed-sign) single-trial coefficients that combine the components (*h*_*n*_^*i* , *j*^ in Eq. (2)), so they can account for negative activations (as depicted for one subject in Fig. 2C).

### Functional role of components via discrimination analyses

To characterize the functional role of each component with respect to the task at hand, we performed discrimination analyses that related the component activations along the stimulus category and stimulus difficulty dimensions on each trial. Most importantly, these analyses served to compare our results with previous findings on the same data in order to validate the proposed method and demonstrate its merits.

**Fig. 4:**
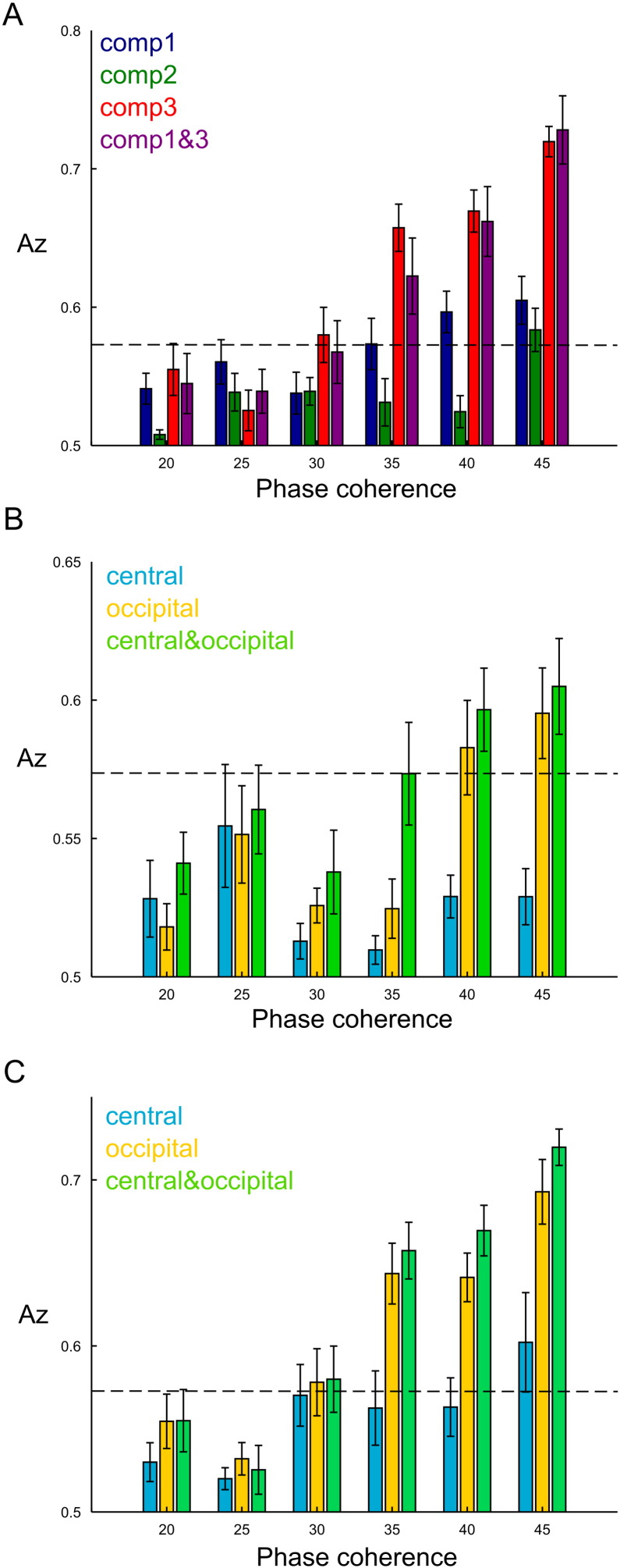
Face versus car discrimination using the space-by-time decomposition of EEG data. A) Average decoding performance across subjects at all phase coherence levels for each temporal component separately (blue, green and red respectively) and when using the first and third temporal components together (magenta). B) Average decoding performance across subjects at all phase coherence levels when combining the first temporal component with each spatial component separately (cyan and yellow respectively) and when using the two spatial components together (light green). C) Average decoding performance across subjects at all phase coherence levels when combining the third temporal component with each spatial component separately (cyan and yellow respectively) and when using the two spatial components together (light green). Dashed lines indicate significance levels.

First, we tested whether the component activations allowed discrimination between the stimulus categories (face or car). We employed an LDA algorithm to perform a face versus car discrimination at each phase coherence level separately using the signed coefficients **H**_*n*_ to represent each trial. We first examined the discrimination performance of each of the three temporal components (Fig. 4A). To compute this, we used as input to LDA for each temporal component the two coefficients combining it in each trial with the two spatial components. We found that faces from cars were best discriminated by the third temporal component (red bars) (Fig. 4A). The discrimination power of the third component dropped as phase coherence decreased and fell below chance level for the last three phase coherence levels. The first temporal component (blue bars) also discriminated faces from cars above chance at the highest two phase coherence levels, whereas the second did not (green bars). Interestingly, combining the first and third temporal components (purple bars) did not yield higher discrimination than the third component alone, which indicates that almost all category information was carried by the third component. These findings suggest that the third component is more closely related to the actual perceptual decision made by the subjects, which is consistent with previous work showing that a late EEG component (around 300 ms post-stimulus) was more predictive of performance than an earlier one (around 170 ms post-stimulus) (Philiastides et al., 2006, Philiastides and Sajda, 2006a, Philiastides and Sajda, 2006b, Philiastides and Sajda, 2007, Ratcliff et al., 2009).

Next, we examined the contribution of each spatial component to face versus car discrimination. To do this, we weighed each of the temporal components by each spatial component in turn. In Figs. 4B-C, we report discrimination performance in space for the first and third temporal component separately (Figs. 4B and C respectively). In both cases, the second spatial component had significantly higher discrimination performance than the first one and the combination of the two did not yield significantly higher discrimination than the second spatial component. Hence, the occipitoparietal electrode sites carried most of the category information either around 150 ms or around 450 ms post-stimulus. This finding is consistent with earlier work investigating the spatial and temporal characteristics of perceptual categorizations using EEG-informed fMRI measurements. In particular, the N170 EEG component (our first temporal component) was shown to originate at the fusiform face area (FFA) and the face-selective P300 EEG component (our third temporal component) at the lateral occipital complex (LOC) (Philiastides and Sajda, 2007).

**Fig. 5:**
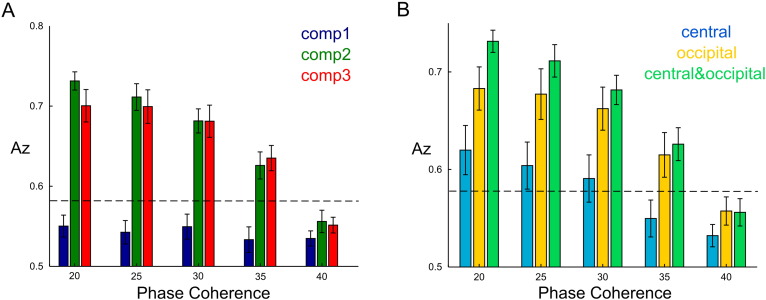
Phase coherence discrimination using the space-by-time decomposition of EEG data. A) Average decoding performance across subjects contrasting the highest phase coherence level (45%) with all other levels for each temporal component separately (blue, green and red respectively). B) Average phase coherence decoding performance across subjects when combining the second temporal component with each spatial component separately (cyan and yellow respectively) and when using the two spatial components together (light green). Dashed lines indicate significance levels.

We also investigated whether the space-by-time decomposition of EEG data allowed discriminating between phase coherence levels. To test this, we pooled together all face and car trials from each coherence level and then employed LDA to perform pairwise classifications between the highest phase coherence level and all the other levels. As we did before for the face versus car discrimination, we first examined discrimination performance of each temporal component (by collapsing across the spatial components). The second temporal component showed the highest discrimination in this task and was followed by the third component, whereas the first component did not reliably discriminate between phase coherence levels (Fig. 5A). As expected, discrimination of the second and third component decreased for phase coherence levels nearer one another.

When examining the spatial structure of discrimination performance of the second temporal component, we found that the occipitoparietal spatial component had the highest discrimination power (Fig. 5B). The central spatial component also discriminated above chance for most phase coherence comparisons, while the combination of the two spatial components further increased the discrimination performance suggesting that the two spatial components carried partly complementary information about phase coherence. This result agrees with previous work implicating the second temporal component with attentional control and allocation of resources for more difficult trials in brain regions corresponding to the two identified spatial components (anterior cingulate cortex(ACC; BA 32) and parietal cortex-intraparietal sulcus (IPS)) (Philiastides et al., 2006, Philiastides and Sajda, 2007).

### Performance comparison with sliding-window LDA analysis

**Fig. 6:**
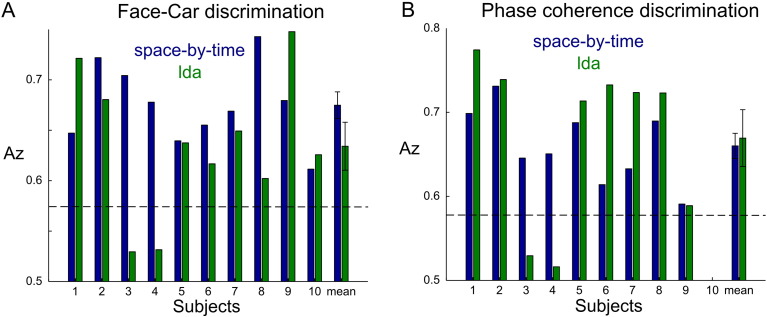
Decoding performance comparison between the space-by-time decomposition and LDA in a temporal window. A) Face versus car decoding of the third temporal component of the space-by-time decomposition (blue) compared to decoding performance obtained by LDA in the same temporal window (green). Reported values are averages across the three significant coherence levels for all subjects. Rightmost bars are the grand averages (± sem) across subjects for the two methods. A) Phase coherence decoding of the second temporal component of the space-by-time decomposition (blue) compared to decoding performance obtained by LDA in the same temporal window (green). Reported values are averages across the four significant coherence levels for all subjects. Rightmost bars are the grand averages (± sem) across subjects for the two methods. Dashed lines indicate significance levels.

To assess the merits of the space-by-time representation in terms of discrimination performance, we compared our results with the ones obtained with a sliding-window LDA approach applied to the raw EEG data, i.e. not preceded by any dimensionality reduction in either space or time. We defined the temporal windows so as to correspond to the three temporal components identified here (see Materials and Methods). Fig. 6 shows the discrimination performance comparison between the two methods across all subjects. In Fig. 6A, we report face versus car decoding performance of the third temporal component averaged across the three phase coherence levels that showed above chance discrimination with both methods. The space-by-time representation achieved higher discrimination on average (though not significantly different from the LDA analysis) and had more robust performance on a subject-by-subject basis. In particular, the space-by-time representation achieved consistent above chance discrimination performance for all subjects (blue bars). On the other hand, sliding-window LDA achieved higher discrimination relative to the current method in three of the ten subjects but also had below chance categorization for two subjects (green bars). Overall, the subject-by-subject variability in face versus car decoding performance of the space-by-time decomposition was significantly lower compared to the sliding window LDA analysis (p = 0.0465, F-test for equal variances). Regarding phase coherence level discrimination, we compared performance of the two methods using the second temporal component and averaged across the four phase coherence comparisons that showed above chance discrimination (Fig. 6B). Similarly in this case, there was no significant difference between the two methods at the population level. The space-by-time decomposition performed consistently above chance for all subjects also in this case. The sliding-window LDA approach superseded the space-by-time decomposition in six of the nine subjects (the tenth subject had no second temporal component) but as before it had below chance performance in two subjects. Again, when comparing the decoding performance variability of the two methods for phase coherence discrimination, we found that it was significantly lower for the space-by-time decomposition (p = 0.0332, F-test for equal variances). Overall, these findings suggest that the space-by-time decomposition was successful in extracting condition-relevant activity consistently well across the group.

## Discussion

In this study, we developed and validated a novel computational approach for the single-trial analysis of time-series neuroimaging data. We introduced a new algorithm to decompose high-dimensional time-varying encephalographic activity into a small number of spatial and temporal components. By means of a decoding analysis, we showed that the extracted components correspond to distinct neural signatures of experimental parameters, such as the presented stimulus and task difficulty.

### Foundations, properties and usability of the method

The proposed space-by-time decomposition relies on non-negative matrix factorization, a dimensionality reduction method that has gained wide interest in recent years (Lee and Seung, 1999). Our method inherits the advantages of NMF decompositions, i.e. it yields low-dimensional representations that correspond to meaningful parts of the recorded neural activity.

Another development of our method is the incorporation of cluster-NMF in the component extraction algorithm, which serves to make space-by-time NMF applicable to signed data. Hence, the new method inherits the sparseness and clustering properties of cluster-NMF which translate into the extraction of highly interpretable components (Ding et al., 2010). Specifically, the spatial components have sparse activations of sensors and thus may correspond to clusters of sensors that record neural activity from brain regions with similar function. Equivalently, the temporal components have sparse activations in time, i.e. they comprise single bumps of activity that may correspond to temporal windows in which a specific brain function is completed.

Here, we also extended the standard two-factor NMF to a tri-factorization that uncovers both the spatial and the temporal structure of the recorded signals (Delis et al., 2014). Our space-by-time M/EEG decomposition offers a compact and crisp description of the recorded EEG signals in both space and time. The resulting representation comprises meaningful spatial and temporal components that not only resemble the original EEG data but also describe reliably all differences between experimental conditions. Importantly, this representation is directly obtained from the data in an unsupervised manner, i.e. without the need to contrast conditions, but succeeds in summarizing all differences between conditions in a single decomposition.

One of the reasons for the good performance of the new algorithm was the enforcement of two different objective functions for the optimization of a) the spatial and temporal components and b) the single-trial activation coefficients. This double objective function aimed at achieving simultaneously two goals: a) extracting sparse non-overlapping spatial and temporal components that can be interpreted as serving distinct neural functions and b) approximating the recorded EEG signals as accurately as possible in single-trials using a small set of coefficients. However, the use of two objectives also has a disadvantage, namely that convergence of the algorithm to a local optimum is not guaranteed. While this was not a problem with the dataset analysed here, users of this algorithm may encounter convergence issues in other datasets. To alleviate this problem, we recommend performing multiple runs of the algorithm with different (e.g. random) initializations and selecting the algorithm output that has the highest discrimination power.

### Comparison with other approaches

Typically, unsupervised learning techniques such as ICA or PCA are used to reduce the dimensionality of time-series neuroimaging data in only one of the two dimensions, i.e. they identify only temporal or (more often) only spatial components of the data (Friston et al., 1993, Makeig et al., 1996). Also, these methods are based on assumptions of orthogonality (PCA) or independence (ICA) which usually lead to holistic representations of the data. Hence, they often struggle to identify components that are condition-specific and not orthogonal/independent to others. In contrast, we showed here that our NMF-based decomposition succeeds in identifying components that have distinct functional roles and describe different aspects of information carried by the EEG data.

An alternative to PCA and ICA that operates in both space and time is Parallel Factor Analysis (PARAFAC) (Maris et al., 2011, Miwakeichi et al., 2004, Morup et al., 2006, van der Meij et al., 2012). PARAFAC identifies unique components that comprise both a spatial and a temporal signature (thus same number of spatial and temporal dimensions), which however results in a less flexible representation of the data than our space-by-time factorization. Our decomposition, instead, identifies separate spatial and temporal components (of possibly different dimensionality) that are multiplexed in single trials, which allows the same temporal component to be combined with different spatial ones (and vice-versa) in order to encode different properties of the dataset. For example, we showed in this study that the same spatial component (comprising mainly occipitoparietal electrodes) a) discriminates faces from cars and b) encodes noise levels in different temporal windows (150/450 ms and 250 ms respectively). Also, one temporal component (peaking at 250 ms) when combined with two spatial components (an occipital and a central one) together achieves higher decoding performance than with each one of them separately.

The above limitation of PARAFAC is overcome by the family of Tucker decompositions that allow different numbers of components to be combined using a core tensor (Cichocki et al., 2015, Cong et al., 2015) and can incorporate non-negativity as well as other types of constraints (Andersson and Bro, 2000, Cichocki et al., 2008, Phan and Cichocki, 2011). Our method differs from the Tucker decompositions in terms of both its algorithmic foundations and its applicability. From a methodological point of view, the extracted components are optimized to achieve different objectives in the two methods (see the cluster-NMF objective function in Eq. (8), (9)). Our method has a unique clustering feature (inherited from the cluster-NMF algorithm (Ding et al., 2010)) that involves constraining the extracted components to be derived from convex combinations of the input data and interpreting them as posterior cluster probabilities (Ding et al., 2010). This clustering constraint yields succinct non-overlapping components with distinct functional roles. To our knowledge, this feature has not been incorporated in the Tucker decompositions.

In addition, the two methods rely on different optimization algorithms ((hierarchical) alternating least squares (Acar et al., 2011, Cichocki and Phan, 2009) or column-row decomposition (Caiafa and Cichocki, 2010) for Tucker decompositions versus multiplicative update rules for scNM3F). We refer to Supplementary Material (Comparison with non-negative Tucker-2 decomposition and Supp. Figs. 2-3) for a formal comparison between our method and non-negative Tucker-2 on the EEG data of our example subject. In brief, we showed that the two methods give different results and this difference demonstrated specific advantages of our new algorithm, such as the extraction of more succinct representations in space and time and the higher decoding power of these representations. By comparing with a version of our algorithm that does not impose clustering constraints, we also demonstrated that these differences are mainly due to the clustering property of the scNM3F algorithm.

PARAFAC and Tucker decompositions have been shown to be effective in decomposing time-frequency or space–time-frequency representations of M/EEG data (Latchoumane et al., 2012, Morup et al., 2007, Phan and Cichocki, 2011). In these applications, the input matrices included nonnegative entries of trial-averaged or condition-labelled data. An advance of our work is that we applied a 3-factor decomposition to signed space–time EEG data, and decode reliably subtle changes in activity from higher-level cognitive states (i.e. decision states) in single trials. Furthermore, here we offered a functional characterization for each spatial and temporal component by relating them to distinct cognitive states during decision making. In contrast, similar approaches have typically been validated using simpler experimental tasks by capitalizing on sensory or motor-related responses that are stronger and easier to obtain.

Another group of techniques that are designed to extract components from M/EEG signals use supervised learning. These methods aim to identify components that discriminate between experimental conditions rather than approximate the recorded data (like unsupervised learning methods). The sliding-window LDA we used here as well as other decoders fall in this category (Parra et al., 2002, Parra et al., 2005, Sajda et al., 2009). These approaches focus on specific temporal windows of the time-varying signals in order to identify discriminating spatial components, hence, in contrast to our method, they do not characterize fully the temporal structure of the data. An extension of these methods that decomposes the data in both space and time is Bilinear Discriminant Component Analysis (BDCA) (Dyrholm et al., 2007), which identifies unique components with a spatial and a temporal signature (like PARAFAC). In contrast to our method, the generalization power of BDCA relies heavily on the addition of smoothness regularization constraints to the extracted spatial and temporal components.

When comparing our new method with a standard supervised learning method (sliding-window LDA) in terms of discrimination performance, we showed that our new method performs as well as sliding-window LDA and gives more robust results across subjects. In fact, sliding-window LDA fails to discriminate between conditions for a few subjects. In contrast, our method performs consistently and significantly above chance across all participants. The consistency of our method likely derives from the effectiveness of the proposed decomposition in capturing all the condition-discriminating information of the EEG signals and suppressing condition-irrelevant signal variations. In particular, our approach is designed to incorporate two steps that accomplish complementary objectives: it firstly learns a low-dimensional subspace (information extraction) and then, it uses this information to discriminate between conditions (classification) (Delis et al., 2013a, Delis et al., 2013b). As indicated by our results, the space-by-time decomposition succeeds in identifying a subspace on which all condition-relevant differences are preserved and attributed to distinct dimensions. Hence, classification on this subspace is more robust than on the raw data space which includes far more dimensions that are also corrupted by condition-irrelevant variability.

### Neural interpretation and functionality of the components

In this study, we found that the whole-scalp time-varying EEG activity during the performance of a perceptual categorization task can be described by two spatial and three temporal components. Our findings suggest that, in time, there is a sequence of three neural events/processes, which has also been reported in previous studies (Philiastides et al., 2006, Philiastides and Sajda, 2006a, Philiastides and Sajda, 2006b). The sequence of unsigned temporal components represents this sequence of neural processes, i.e. “when” they take place. In space, we find groups of sensors that co-vary to encode these processes in different brain regions. By keeping them unsigned, we signify that they represent functional groupings of sensors, i.e. “where” the processes take place. Then, to characterize the strength of each process at each time point and spatial location, we use the signed coefficients to signify the polarity of the evoked response (i.e. either positive or negative).

The resulting space-by-time representation uses a small number of parameters (2 × 3 = 6 single-trial coefficients) to characterize the EEG activity on each individual trial. By mapping these single-trial coefficients onto the stimuli presented in each trial, we demonstrated that the extracted component activations relate reliably to differences between experimental conditions. Hence, we validated that the extracted components encode experimental parameters, showed that each component has a distinct functional role and uncovered the combinations of components that carry most of the neural information about the task. Specifically, we showed that the activation of occipitoparietal sensors 150 ms and 450 ms post-stimulus discriminates between face and car images and the activation of occipital and central sensors 250 ms post-stimulus encode the noise level of the presented images. These findings are consistent with the results reported in previous studies using a subset of these data (Philiastides et al., 2006, Philiastides and Sajda, 2006a, Philiastides and Sajda, 2006b, Ratcliff et al., 2009) as well as an EEG-informed fMRI study that localized the spatial signatures of the extracted temporal components (Philiastides and Sajda, 2007). Also, when comparing the identified decompositions across subjects, we found that the components were highly reproducible. This result supports the credibility of the extracted spatial and temporal components as reliable descriptors of where and when the neural processes underlying this categorization task take place.

We also suggest that our method can be proven useful in addressing open questions relating to a recent debate regarding the neuronal origins of the EEG signal. Recent studies using direct measures of EEG and spiking activity (Whittingstall and Logothetis, 2009) and indirect comparisons of their stimulus selectivity (Ng et al., 2013) – together with theoretical considerations (Mazzoni et al., 2010) – demonstrated that, on average, the EEG robustly reflects the strength of the mass spiking multi-unit activity of pyramidal neurons both in the power of gamma EEG oscillations (the higher the power, the larger the underlying spike rate) and - most prominently - in the phase of low-frequency EEG oscillations.

In particular, variations in phase at a single spatial location may correspond to variations of the firing rate at that spatial location around its mean rate (i.e. relative to baseline firing rate) that might be reflected as changes in the sign of the EEG signal. Although determining whether positive values and negative values correspond to a decrease or increase of firing remains difficult, in principle it can be determined by checking whether positive or negative values of our decomposition coefficients at a given spatial location correspond to higher or lower values of EEG gamma power, as the latter correlates robustly and positively to spike rate (Mazzoni et al., 2010, Whittingstall and Logothetis, 2009). These predictions can be quantitatively tested in future studies where our method can be applied to the analysis of direct recordings of EEG and spiking activity.

### Conclusion

We believe that the properties of the space-by-time M/EEG decomposition we described above, i.e. its simplicity, condition-relevance, functionality and consistency, can make it a useful tool for the single-trial analysis of multichannel time-varying M/EEG activity in a variety of experimental tasks. In particular, we suggest that it can be especially effective when the aim is to a) tease apart condition-dependent neural patterns with different functional roles, b) reveal their spatial and temporal representations and c) quantify their relative contribution to discrimination between experimental conditions.

## Appendix A Sample-based non-negative matrix tri-factorization algorithm (sNM3F)

The sNM3F algorithm takes as input a strictly non-negative data matrix **M** and estimates non-negative spatial modules **W**^spa^, temporal modules **W**^tem^ and activation coefficients **H**. The algorithm iteratively updates **W**^spa^, **W**^tem^ and activation coefficients **H** using the multiplicative update rules given in Eqs. (11), (12), (13) below.

### A.1 Algorithm formulation

By convention, we represent a trial-dependent matrix **A** as a vertical concatenation of all the single-trial matrices, that is

$$A=\left(\begin{array}{c} A^{1}\\ A^{2}\\ \vdots \\ A^{n} \end{array}\right).$$

We define the block transpose operation, denoted by the symbol “prime”, which transforms a matrix **A** with vertical concatenation of the trials to **A**^′^ with horizontal concatenation of the trials and vice-versa. Thus, **A**^′^ = (**A**^1^ **A**^2^ … **A**^*n*^) and (**A**^′^)^′^ = **A**.

The sNM3F algorithm uses the above operation to reshape the elements of the trial-dependent matrices, such as **M** and **H**. The final form of the algorithm is given below:

1) Initialize **W**_*tem*_(*T* × *P*), **H**^′^(*P* × *LN*), and **W**_*spa*_(*L* × *S*)with random entries
2) Given **H**^′^, **W**^tem^ and the data matrix **M**(*TN* × *S*), update **W**_*spa*_:
  a. Compute **G**^′^ = **W**_*tem*_**H**^′^
  b. Block transpose **G**^′^ → **G**
  c. Update **W**_*spa*_ to improve the approximation **M** ≃ **GW**_*spa*_, i.e. for all *i* ∈ {1, ... , *L*} , *j* ∈ {1, ... , *S*}:
    (11) $$W_{\mathrm{spa}}^{i,j}\leftarrow W_{\mathrm{spa}}^{i,j}{\left(G^{T}\mathrm{GM}\right)}^{i,j}/{\left(G^{T}GW_{\mathrm{spa}}\right)}^{i,j}.$$
3) Given **H**^′^, **W**_*spa*_ and **M**, update **W**_*tem*_:
  a. Block transpose **H**^′^ → **H** and **M** → **M**^′^
  b. Compute **V** = **HW**_*spa*_
  c. Block transpose **V** → **V**^′^
  d. Update **W**_*tem*_ to improve the approximation **M**^′^ ≃ **W**_*tem*_**V**^′^, i.e. for all *i* ∈ {1, ... , *T*} , *j* ∈ {1, ... , *P*}:
    (12) $$W_{\mathrm{tem}}^{i,j}\leftarrow W_{\mathrm{tem}}^{i,j}{\left(M^{\prime }V^{\prime }\right)}^{i,j}/{\left(W_{\mathrm{tem}}{V^{\prime }}^{T}V^{\prime }\right)}^{i,j}.$$
4) Given **W**_*tem*_ and **W**_*spa*_, update **H**:
  a. For all *n* ∈ {1, ... , *N*}, update **H**_*n*_ to improve the approximation **M**_*n*_ ≃ **W**_*tem*_**H**_*n*_**W**_*spa*_, i.e. for all *i* ∈ {1, ... , *P*} , *j* ∈ {1, ... , *L*}:
    (13) $$H_{n}^{i,j}\leftarrow H_{n}^{i,j}{\left({\left(W_{\mathrm{tem}}\right)}^{T}M_{n}{\left(W_{\mathrm{spa}}\right)}^{T}\right)}^{i,j}/{\left({\left(W_{\mathrm{tem}}\right)}^{T}W_{\mathrm{tem}}H_{n}W_{\mathrm{spa}}{\left(W_{\mathrm{spa}}\right)}^{T}\right)}^{i,j}.$$
5) If decrease in approximation error $\sum_{n=1}^{N}{\left\|M_{n}-W_{\mathrm{tem}}H_{n}W_{\mathrm{spa}}\right\|}^{2}$ is below a given tolerance, stop. Otherwise, go to step 2.

## Appendix B Supplementary data

Supplementary material. Image 1
