# Supplementary material for: Space-by-time decomposition for single-trial decoding of M/EEG activity
Source: Neuroimage. 2016 Jun;133:504–15. doi: 10.1016/j.neuroimage.2016.03.043 (PMC4907687; doi:10.1016/j.neuroimage.2016.03.043)
Supplement: Supplementary file 1 — Supplementary material. [file mmc1.pdf]

Supplementary Material

Space-by-time decomposition for single-trial decoding of M/EEG activity

Ioannis Delis[a,b], Arno Onken[c], Philippe G. Schyns[a], Stefano Panzeri[c] and Marios G. Philiastides[a]

[a]Institute of Neuroscience and Psychology, University of Glasgow, Glasgow G12 8QB, United Kingdom

[b]Department of Biomedical Engineering, Columbia University, New York, NY 10027, USA.

[c]Neural Computation Laboratory, Center for Neuroscience and Cognitive Systems@UniTn, Istituto Italiano di Tecnologia, Via Bettini 31, 38068 Rovereto (TN), Italy

Comparison with non-negative Tucker-2 decomposition

Here we offer a formal comparison between our method and Tucker-2 decomposition. To implement the Tucker-2 decomposition, we used the N-way Matlab toolbox (Andersson and Bro, 2000) and imposed non-negativity constraints to the spatial and temporal components and no constraints to the core tensor.

First, we applied the Tucker-2 decomposition to the data of our example subject and compared the extracted components with the ones identified by our approach. We illustrate the results in Supp. Figure 2A-B. The temporal and spatial components extracted by the two methods have considerable differences. In particular, Tucker-2 merges the first two temporal components into a single one and the other two components overlap highly. A high overlap is also observed for the two spatial components. Instead, scNM3F yields succinct non-overlapping temporal and spatial components, which as we showed in the paper encode different cognitive functions.

ssNM3F algorithm. These differences could be due to the clustering feature of our method that is not included in Tucker decompositions or the difference in the optimization algorithm (multiplicative update rules for our method versus alternating least squares for Tucker decompositions). To investigate these two alternatives, we also compared our results with another NMF-based algorithm that does not impose clustering constraints. We built this algorithm by extending semi-NMF (Ding et al., 2010) to a 3-factor decomposition and named it sample-based semi-nonnegative matrix tri-factorization (ssNM3F). For $\mathbf{W}_{tem}$ and $\mathbf{W}_{spa}$ the update rules of semi-sNM3F are:

$$\mathbf{W}_{tem}^{i,j} \leftarrow \mathbf{W}_{tem}^{i,j} \sqrt{\frac{[(\mathbf{M}_{tem}\mathbf{G}_{tem}^{T})^{+}]^{i,j} + [\mathbf{W}_{tem}(\mathbf{G}_{tem}\mathbf{G}_{tem}^{T})^{-}]^{i,j}}{[(\mathbf{M}_{tem}\mathbf{G}_{tem}^{T})^{-}]^{i,j} + [\mathbf{W}_{tem}(\mathbf{G}_{tem}\mathbf{G}_{tem}^{T})^{+}]^{i,j}}} \qquad (1)$$

$$\mathbf{W}_{spa}^{i,j} \leftarrow \mathbf{W}_{spa}^{i,j} \sqrt{\frac{[(\mathbf{M}_{spa}^{T}\mathbf{G}_{spa})^{+}]^{i,j} + [\mathbf{W}_{spa}^{T}(\mathbf{G}_{spa}^{T}\mathbf{G}_{spa})^{-}]^{i,j}}{[(\mathbf{M}_{spa}^{T}\mathbf{G}_{spa})^{-}]^{i,j} + [\mathbf{W}_{spa}^{T}(\mathbf{G}_{spa}^{T}\mathbf{G}_{spa})^{+}]^{i,j}}} \qquad (2)$$

Where $\mathbf{G}_{tem}$ is the reshaped version of $\mathbf{H}_{tem}\mathbf{W}_{spa}$ with dimension ($P \times SN$) and $\mathbf{G}_{spa}$ is the reshaped version of $\mathbf{W}_{tem}\mathbf{H}_{spa}$ with dimension ($TN \times L$). $\mathbf{H}_{tem}$ and $\mathbf{H}_{spa}$ are reshaped versions of the coefficient matrix $\mathbf{H}$ with dimensions ($PN \times L$) and ($P \times LN$) respectively. Each $\mathbf{H}_n$ is iteratively updated for all $n \in \{1,...,N\}$ using the same rule as in scNM3F:

$$\mathbf{H}_n \leftarrow \mathbf{W}_{tem}^{-1}\mathbf{M}_n\mathbf{W}_{spa}^{-1} \qquad (3)$$

Importantly, ssNM3F is devised in order to be applied to signed data but does not have the clustering feature of scNM3F.

Importance of clustering feature. By applying ssNM3F to the EEG data of the example subject, we found that, similarly to the Tucker decomposition, it identifies highly-overlapping temporal and spatial components (Supp. Figure 2C). This observation suggests that the observed differences in the extracted components are mainly due to the clustering feature of the scNM3F algorithm. We also compared the discrimination performance of the three methods on the same data. We found that for both face versus car classification and phase coherence level classification, scNM3F performed better than Tucker-2 and ssNM3F (Supp. Figure 3).

Importance of optimization algorithm. We then examined whether the use of different optimization algorithms may also affect the decomposition outputs. An important difference in this respect is that the update rules used by the two NMF-based algorithms (Eq.4-5 for scNM3F and Supp. Eq. 1-2 for ssNM3F) make use of both the positive and the negative entries of the input data matrix in order to identify components, whereas the alternating least squares algorithm used in the Tucker decomposition relies on a half-wave rectification of the input data, i.e. it ignores the negative entries.

To investigate how this affects the decomposition outputs, we applied ssNM3F and Tucker-2 to simulated data with known ground-truth components. We generated three temporal components as sums of three Gaussian bursts and two spatial components that were gamma distributed and combined those using normal random coefficients (Supp. Figure 4A). We applied the Tucker-2 decomposition and the ssNM3F algorithm to the simulated data and extracted the spatial and temporal components shown in Supp. Figure 4B-C. ssNM3F extracted temporal and spatial components that were more similar to the original ones than Tucker-2. To quantify this, we computed the mean squared error between the original and extracted modules of the two methods over 100 repetitions of data generation and module extraction. We found that the temporal modules identified by ssNM3F were significantly more similar to the original ones ($p<0.001$, t-test) than Tucker-2 and also the spatial modules were slightly but not significantly more similar (Supp. Figure 5). This result suggests that, besides the clustering feature, also the use of multiplicative update rules that take into account the negative entries of the data gives a data reconstruction advantage to the space-by-time decomposition when compared to Tucker-2.

Decoding performance comparison. Finally, we compared the decoding performance of scNM3F and non-negative Tucker-2 on the real data of all 10 subjects (Supp. Figure 6). We found that our method performed significantly better than the Tucker-2 decomposition at the

population level for both face versus car classification and phase coherence level classification ($p<0.01$, t-tests).

Supplementary Figures

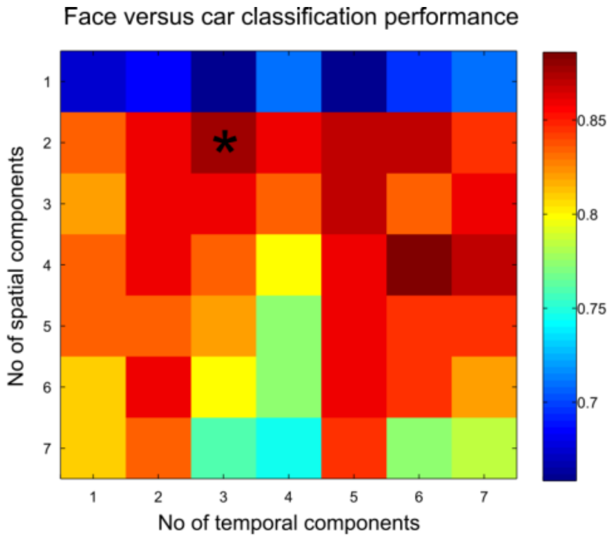

Supp. Figure 1: Dependence of face versus car classification performance (for the highest phase coherence level) on the number of spatial and temporal components. Classification peaks at 2 spatial, 3 temporal components (indicated by a star) and shows no further increase for larger numbers of components. Hence, we selected this set of components for all further analyses.

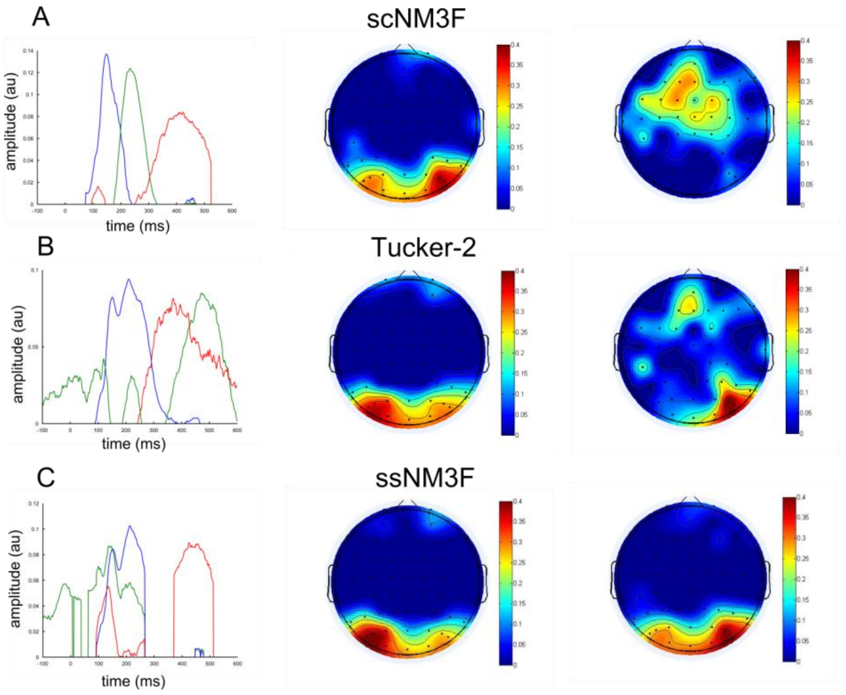

Supp. Figure 2: Comparison of the output of the proposed scNM3F algorithm (A) with the non-negative Tucker-2 decomposition (B) and the ssNM3F algorithm (an NMF-based algorithm that does not have the clustering feature) (C) on the EEG data of the example subject.

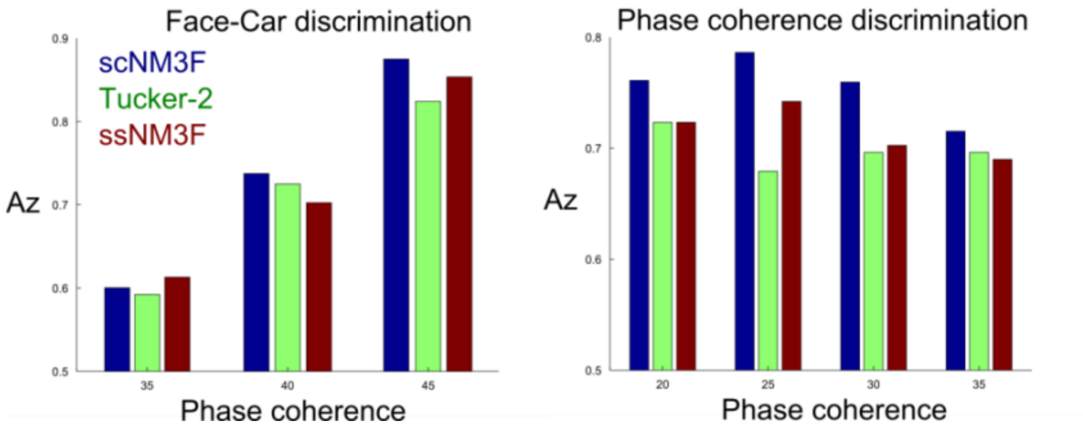

Supp. Figure 3: Comparison of scNM3F (blue) with the non-negative Tucker-2 decomposition (green) and ssNM3F (red) in terms of their performance on face versus car classification (left) and phase coherence classification (right) for all significant phase coherence levels.

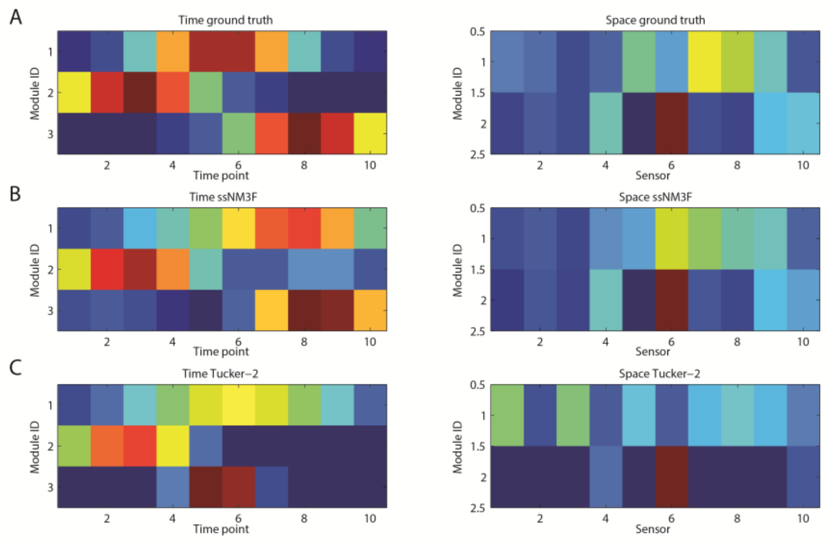

Supp. Figure 4: Comparison of the components extracted by ssNM3F and non-negative Tucker-2 decomposition on simulated data. A) The simulated temporal and spatial components. B) The temporal and spatial components identified by ssNM3F. C) The temporal and spatial components identified by non-negative Tucker-2.

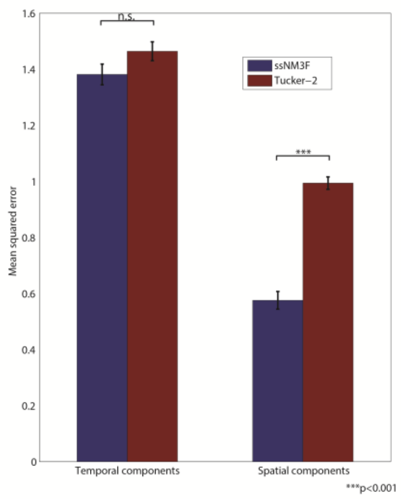

Supp. Figure 5: Comparison of ssNM3F and non-negative Tucker-2 decomposition in terms of component reconstruction on simulated data. Colored bars indicate mean squared errors between the original components and the ones identified by ssNM3F (blue) and Tucker-2 (red). Data generation and component extraction were repeated 100 times. Error bars represent standard error means.

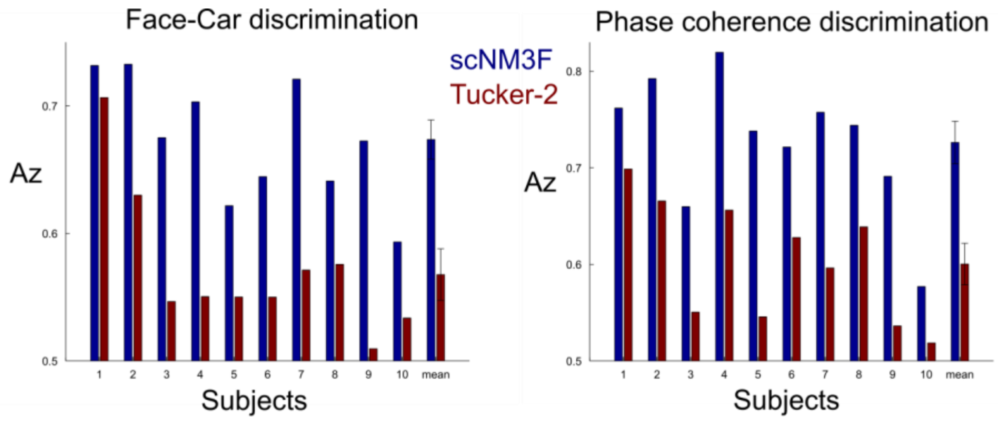

Supp. Figure 6: Decoding performance comparison across subjects between the space-by-time decomposition and the Tucker-2 decomposition. A) Face versus car decoding. Reported values are averages across the three significant coherence levels for all subjects. Rightmost bars are the grand averages (±sem) across subjects for the two methods. A) Phase coherence decoding. Reported values are averages across the four significant coherence levels for all subjects. Rightmost bars are the grand averages (±sem) across subjects for the two methods.

Supplementary References

Andersson, C.A., Bro, R., 2000. The N-way Toolbox for MATLAB. Chemometrics and Intelligent Laboratory Systems 52, 1-4.

Ding, C., Li, T., Jordan, M.I., 2010. Convex and semi-nonnegative matrix factorizations. IEEE Trans Pattern Anal Mach Intell 32, 45-55.
